# Benefits and Challenges of California Offshore Wind Electricity: An Updated Assessment

**DOI:** 10.3390/en18010118

**Published:** 2024-12-31

**Authors:** Adam Rose, Nathaniel Gundersen, Yamini Kumar, Joshua Jacobs, Isabel Reynoso, Najmedin Meshkati

**Affiliations:** 1 Price School of Public Policy, University of Southern California, Los Angeles, CA 90089, USA; 2 Viterbi School of Engineering, University of Southern California, Los Angeles, CA 90089, USA

**Keywords:** offshore wind electricity, technology assessment, benefits, co-benefits, challenges, california coast, policy recommendations

## Abstract

Offshore wind (OSW) technology has recently been included in California’s plans to achieve 100% carbon-free electricity by 2045. As an emerging technology, many features of OSW are changing more rapidly than established renewable options and are shaped by local circumstances in unique ways that limit transferrable experiences globally. This paper fills a gap in the literature by providing an updated technological assessment of OSW in California to determine its viability and competitiveness in the state’s electricity generation mix to achieve its near-term energy and environmental goals. Through a critical synthesis and extrapolation of technical, social, and economic analyses, we identify several major improvements in its potential. First, we note that while estimates of OSW’s costs per MWh of installed capacity have generally documented and projected a long-term decline, recent technical, microeconomic, and macroeconomic factors have caused significant backsliding of this momentum. Second, we project that the potential dollar value benefits of OSW’s greenhouse gas reduction capabilities have increased by one to two orders of magnitude, primarily due to major upward revisions of the social cost of carbon. Several co-benefits, including enhanced reliability, economic growth, and environmental justice, look to be increasingly promising due to a combination of technological advances and policy initiatives. Despite these advancements, OSW continues to face several engineering and broader challenges. We assess the current status of these challenges, as well as current and future strategies to address them. We conclude that OSW is now overall an even more attractive electricity-generating option than at the beginning of this decade.

## 1. Introduction

Offshore wind (OSW) electricity is a recent entrant into the mix of technologies to address energy and climate change issues. Although there are only three online OSW installations in the U.S. at present, and formal private and public sector planning for them did not become prominent until the beginning of this decade, this unique technology is being touted as an important part of the future renewable energy technology mix (see, e.g., [1,2]).

Globally, OSW is also expected to play a valuable role with respect to renewable energy production. A total of 115 countries possess approximately 71 TW of technically extractable energy supply, with the U.S. providing an estimated 7.4% of total potential [3]. Total installed capacity reached 75 GW at the end of 2023, and the industry has experienced large increases in deployment and total capacity potential in the pipeline of installed and planned projects in recent years [4–6]. While Europe and China lead the world in OSW development, the U.S. is making progress as well.

Across the U.S. and the world, diverse societal, geographical, and technical factors shape the development path of OSW in distinct ways [7–9]. As such, there are differences with respect to local circumstances, and there is a need to understand how these dimensions influence OSW development. This paper specifically examines the California context, which is unique for several reasons, including but not limited to its deep ocean locations for OSW, the concerns of Indigenous communities, and the need for high levels of additional transmission investment. Although there are some limited globally transferrable experiences, our analysis focuses primarily on studies related to California.

Beginning in the year 2020, the senior author of this paper led a major study on the benefits of OSW in California that emphasized several attractive features [10,11]. These included the direct benefits of providing relatively inexpensive electricity and the reduction of greenhouse gases (GHGs). It also included co-benefits relating to improved electricity system reliability, reduction in ordinary air pollutants, reduction in land-based environmental impacts, job creation, equity and environmental justice gains in both urban and rural areas, and California’s potential leadership in OSW manufacturing support service industries. Furthermore, the study identified several challenges to be addressed, including the need for new transmission lines, expanded seaport and cargo vessel capacity, supply chain limitations, and concerns relating to ambient and ocean environments, wildlife, the fishing industry, the military, and decommissioning. The overall conclusion was that the benefits and co-benefits were potentially sizable and that the challenges are tractable with cooperation between the private sector, government at various levels, and local communities. (The original paper was based on an independent study under the auspices of the University of California’s Schwarzenegger Institute for State and Global Policy and was funded by The Energy Foundation, American Clean Power Association (ACP) and Offshore Wind California (OWC). It included input from researchers at leading institutions studying OSW, such as the National Renewable Energy Laboratory (NREL) and Humboldt State University).

New technologies change rapidly, so an updated technology assessment every few years is warranted. Our purpose is to fill a gap in the literature by providing an updated assessment of progress toward California OSW being a viable and competitive renewable electricity generating technology. We accomplish this by providing a critical synthesis of the literature together with interviews with experts in several domains related to OSW.

The next section examines the direct benefits of OSW, which include new projections for declining lifecycle costs and estimates of the increasing societal benefits of GHG reductions. This is followed by a discussion of co-benefits. The last two sections analyze progress addressing engineering-specific, as well as broader industry, societal, and other challenges. In these sections, we provide a background description of each aspect, its current status, recent changes, and the future outlook.

## 2. Background

In an effort to maximize the use of strong offshore wind currents, OSW manufacturers and developers are continuously developing larger turbines and greater generating capacities, supported by improved seabed and platform foundations. In 1980, wind turbines were around 17 m tall and achieved capacities of roughly 75 kW. Today, OSW turbines can reach up to 250 m and produce anywhere from 15 to 18 MW [4]. In water depths of around 60 m or less, fixed-bottom types include monopile, jacket, and gravity-based foundations [12]. In deeper waters, floating foundations tether the turbines to the ocean floor and include spar-buoy, semi-submersible, and tension-leg platform designs. These engineering feats have enabled turbines to reach deeper waters and access stronger and more consistent winds than ever before. While the industry appears to be converging around a 15 MW turbine capacity, Chinese OEMs are pushing this further with announcements for turbine capacities as high as 26 MW [13].

California has a substantial coastal wind resource base to deploy new OSW farms. There are six areas identified by the California Energy Commission (CEC) for OSW development, ranging from 35.8 to 59.6 GW in potential capacity [2]. Prime development areas are located along the North and South-Central Coast, with existing federal leases for the first round of development off the coast of Humboldt County and Morro Bay. Humboldt County and Morro Bay each have potential generation capacities ranging from 1.6 to 2.7 GW and 2.9 to 4.9 GW, respectively.

Project delays across the U.S. have rendered the nationwide target of 30 GW by 2030 unfeasible. However, long-term outlooks and industry efforts remain steady. There are 174 MW of OSW capacity in operation with a significant project pipeline of over 56 GW in development [14]. With 39 active leases and plans by the Bureau of Ocean Energy Management for future lease sales across the country, OSW is poised to play an increasing role in the nation’s renewable portfolio mix [15].

In California, the CEC [2] released the final version of its AB 525 strategic plan, which provides a comprehensive roadmap for OSW development and the necessary supporting infrastructure [2]. The strategic plan is guided by three interim reports that: (1) establish OSW production targets of 2 to 5 GW for 2030 and 25 GW by 2045, (2) assess economic benefits, (3) coordinate permitting for OSW facilities, and (4) consider strategies to address potential impacts. OSW development is moving forward in five lease areas (two in Humboldt and three in Morro Bay) awarded to five different companies—RWE Offshore Wind Holdings and California North Floating in the Humboldt area, and Equinor Wind US, Golden State Wind, and Invenergy California Offshore in the Morro Bay area [15]. Each company is in various stages of permitting and planning, with significant assessment work still needed before any construction begins. A second OSW lease sale in California is being planned for 2028 [16].

The California state legislature has put forward multiple pieces of legislation to support OSW development efforts in recent years. AB 3, AB 1373, and SB 286 include key provisions to advance California’s OSW strategic plan, build out supporting infrastructure capacity, improve in-state manufacturing and project development, establish a central procurement program for long lead time energy resources, and expedite OSW permitting processes (see [17–19]). The proposed AB 2208 (2023) will provide much-needed funding for California ports and OSW infrastructure, along with a range of other climate-related measures, through a USD 10 billion bond act [20]. At the federal level, the Inflation Reduction Act represents landmark legislation that is catalyzing OSW investments in California and across the country [21]. Collectively, these laws are laying the groundwork to accelerate the emerging OSW industry and to support California in meeting its renewable energy targets of a 100% clean electric grid by 2045.

## 3. Direct Benefits

### 3.1. Value Proposition (Cost Competitiveness)

Potential cost savings from OSW stem from the displacement of higher-cost energy alternatives and reductions in system-wide costs through lower-cost resources. Up until the past year, assessments of OSW cost improvements have been optimistic to the extent of projecting LCOE would decline to below USD 100/MWh of installed capacity by 2030 [4,5]. Subsequently, analysts have pointed to recent, ongoing, and projected future conditions that are not so favorable, with the average LCOE for U.S. OSW projects increasing from USD 85 to USD 140 per MWh [22]. One set of considerations revolves around supply chain issues, some of which contribute to cost increases. These are exacerbated by the Russia–Ukraine War, which has also accelerated European interest in OSW and is bidding up the price of OSW components. Both of these are taking place against the cumulative background of the inflation of recent years and higher interest rates.

Another concern is that OSW cost reductions, especially for the more nascent floating technologies, are highly dependent on economies of scale to lower equipment fabrication costs, as well as associated construction infrastructure costs. This scale is driven by power contract procurement, which for floating OSW, will depend largely on the success of a central procurement program in California. Of course, some of these concerns are contradictory or transitory. The increased European activity will overcome the economies of scale concern. The cumulative effect of inflation has been detrimental, but inflation now seems to be in check and interest rate increases are near their peak (note that many of the complicating factors discussed here pertain to other electricity generating technologies, and thus do not adversely affect OSW’s relative competitiveness). As of this writing, there is a new concern about the likely increase in tariffs to be imposed by the new US presidential administration and ensuing potential trade wars. However, there is no reason to believe that these will be other than temporary.

**Figure 1.**
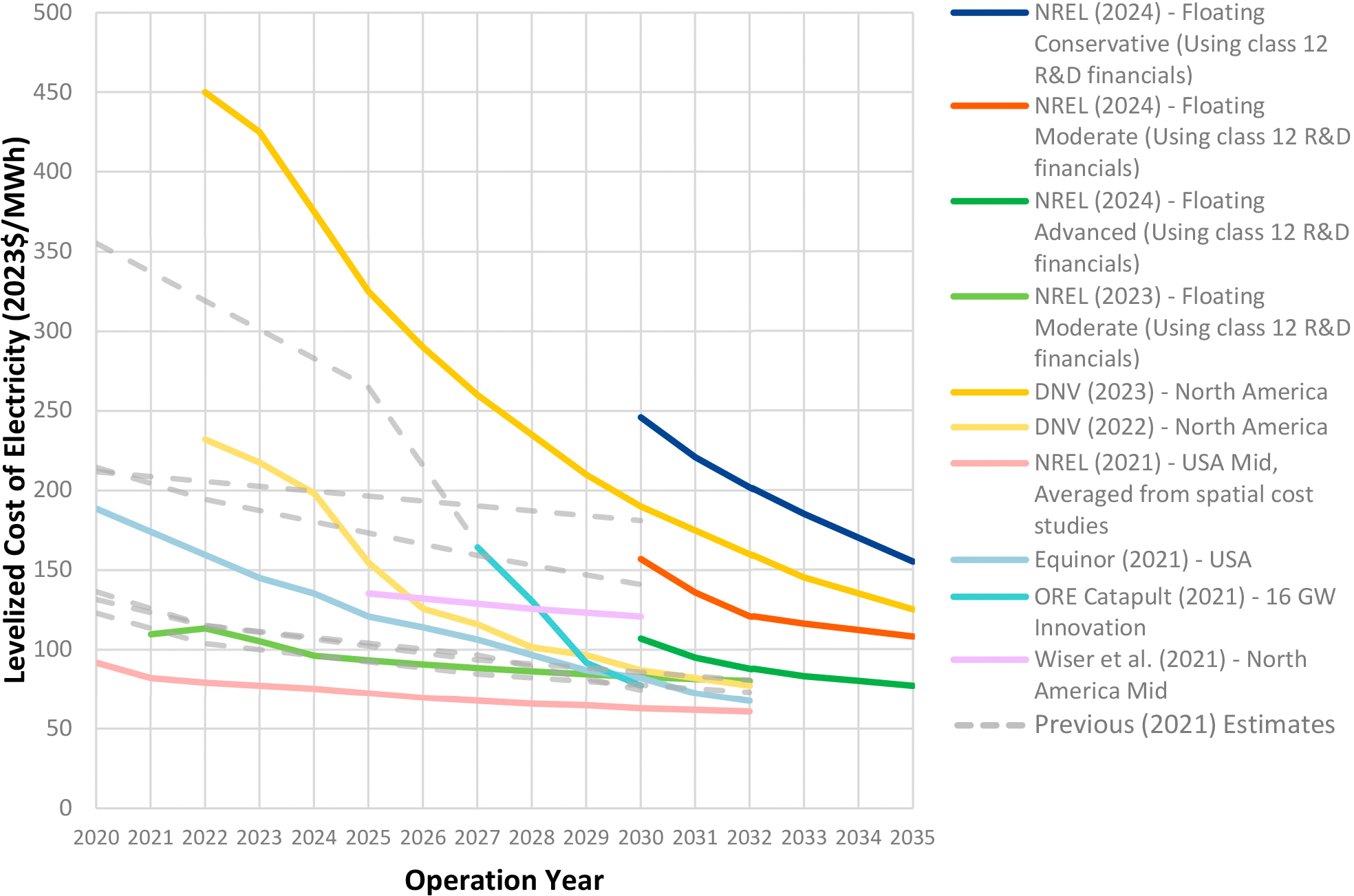
U.S. LCOE Estimates for Floating Offshore Wind Technologies Sources: [23] NREL (2024); [26] NREL (2023); [27] DNV (2023); [28] DNV (2022); [4] NREL (2021); [29] Equinor (2021); [30] ORE Catapult (2021); [31] Wiser et al. (2021). Sources for previous estimates from the original 2022 paper are from top to bottom: [32] ORE Catapult (2018); [33] LBNL (2016)—Floating Median; [34] Musial et al. (2016)—Humboldt Bay; [35] Beiter et al. (2020)—Semi/Morro Bay; [36] Musial et al. (2020)—Semi; [35] Beiter et al. (2020)—Semi/Humboldt.

Given these factors, the current levelized cost of energy (LCOE) has a wide range across projects from USD 75–450/MWh. Up until the past year, several studies anticipated the LCOE of floating OSW falling below USD 100/MWh by 2030 (see Figure 1), whereas the latest projections show a higher range of USD 107/MWh to USD 246/MWh, and not falling below USD 100/MWh until 2035 [23]. As the OSW industry matures, projected cost reductions may stem from several sources including, but not limited to, construction process improvements, utilization of existing supply chains, automated production and standardization of floating substructure fabrication, and additional benefits from higher wind speeds in remote siting areas (see, e.g., [4]). The U.S. Department of Energy’s multi-agency Floating Offshore Wind Shot initiative, which aims to reduce costs 70% by 2035, can also help to accelerate OSW’s cost competitiveness as the industry moves from a pre-commercial to a commercial phase [24].

There are promising signs of increasing cost competitiveness across the U.S., where state governments have generally increased their OSW demand and are developing interstate cooperation frameworks to “stabilize regional markets, lower project costs, and increase economic benefits” [25]. The Inflation Reduction Act (IRA) will continue to play a significant role in offsetting financing limitations and building out supply chains that will support the OSW industry. However, changes to the IRA under the new federal administration could impact the current 30% Production Tax Credit for OSW facilities. Such revisions to the IRA could constrain OSW’s cost competitiveness and create new economic challenges for renewables more broadly.

Moving forward, cost reductions for OSW will be critical relative to the LCOEs of conventional gas-peaking and coal-fired electricity generation. (OSW is now cost competitive with all fossil-fueled electricity generation, except some types of gas combined-cycle operations. It is cost competitive with rooftop and smaller scale community, commercial, and industrial solar electricity. However, it still lags behind onshore wind operations [37]). Additional savings from OSW will also be contingent on competitiveness with other renewables, as well as grid requirements in the future, availability of other resources, and the ability to reach climate goals with a reliable portfolio. The cost of other renewable energy technologies is unlikely to decline as much since they have longer design and operating experiences. Still, the nascent OSW industry stands to garner substantial benefits from increased cost competitiveness and continued innovations in the years ahead.

### 3.2. GHG Reductions

Rose et al. (2022) [10] estimated that 10 GW of OSW would mitigate 1.1 million metric tons of CO_2_ and avoid as much as USD 97.5 million (2023 USD) in climate change damages Where they are listed in this paper, estimates from the original study have been updated from 2019 USD to 2023 USD, using the Consumer Price Index for income estimates, the Producer Price Index for the social cost of carbon, and a GDP deflator of 17.56% for GDP estimates according to [38]. Estimates from the original study have also been adjusted to reflect a 5.1% capacity factor for gas-peaking power plants in California, whereas the original methodology relied on an economy-wide average of 25.7%, which yielded estimates of 4.73 million metric tons of CO_2_ mitigation and USD 436 million in avoided climate change damages. As a result, the updated estimates are relatively conservative in their range of GHG mitigation and benefits potential since capacity factors in other locations are likely to be higher.

The original study assumed reductions in CO_2_ from 10 GW of OSW capacity in California would displace 5 GW of natural gas peaking power plants in the year 2040 (recent estimates of the impact of fixed-bottom OSW along the US Atlantic and Gulf Coasts indicate a similar proportion, where 0.55 MWh of fossil-fueled generation is reduced for every MWh of OSW generation [39]), though they omitted CO_2_ emissions generated along the supply chain from both energy sources. Using a similar methodology and assumptions, the updated reductions of GHGs are generally the same per MWh as the original study, but the estimated benefits have increased substantially (see Table 1). The social cost of carbon (SCC) in 2040 now ranges from USD 170 to USD 270/ton [40], with recent estimates from [41] pushing as high as USD 1,052/ton. Prospects for the size of the development of OSW in California have also increased, raising the scope of potential benefits.

Based on these SCC estimates, the mid-range annual benefit of OSW in 2040 is approximately USD 381 million. This grows to USD 10.7 billion when we consider the benefits from the entire lifecycle of OSW, which is typically 25 years. Should California reach its target of 25 GW by 2045, the annual and lifetime benefits more than double to USD 952 million and USD 26.6 billion, respectively. In the years ahead, the SCC is only expected to rise as population growth and economic activity increase GHG build-up in the atmosphere. Additional GHG reduction may also occur from reductions in OSW’s lifecycle emissions (see Section 5.4 for a discussion of further emissions reductions in the decommissioning phase).

## 4. Co-Benefits

### 4.1. Reliability

OSW plays a critical role in renewable energy portfolios, providing grid-system benefits that facilitate greater grid reliability [10]. Within this section, reliability is used in the narrow sense, referring to the continuous supply of electricity from renewable energy input. We also note the obvious additional contribution to reliability of OSW in terms of diversifying the portfolio of electricity-generating technologies. This differs from more general definitions of the term that relate to any cause of electricity system disruption as defined by the North American Electric Reliability Corporation [42].

OSW has stable intraday capacity factors that are higher on average than onshore wind and solar energy sources, which can stabilize the variable production profiles of other renewables [22]. A major contribution to system reliability is the complementary role that OSW can play with regard to solar electricity. Offshore wind flows peak during early evening hours when net energy demand ramps up quickly, while solar generation typically peaks around noon and vanishes by early evening [43]. This provides a natural balance of electricity flows from these two important sources. OSW peak production periods vary by region, but in California it maximizes in Spring and Summer, aligning with the state’s peak demand while complementing the production profiles for other renewables [43].

Despite concerns about renewables contributing to temporary outages and rolling blackouts in recent years, research has found that renewable portfolios comprised of wind-water-solar electricity and storage maintain grid stability, even under extreme weather conditions [44]. Ongoing technological improvements and the integration of large-scale energy storage systems will further enhance the reliability of OSW energy and ensure a more stable supply of electricity for both OSW and the grid-system as a whole [45]. Storage integration behind-the-meter and at OSW interconnection points can provide additional benefits for the reliability and operations during black-starts [22].

The reliability co-benefits of OSW will also be influenced by grid integration through the development of high-voltage direct current (HVDC) transmission systems and better interconnection points [46]. Altogether, the addition of OSW to renewable energy inputs, and the infrastructure systems to support them, will be crucial for balancing supply and demand, smoothing the “duck curve”, and creating a clean, reliable energy grid-system.

### 4.2. Estimates of Reduction in Major Ordinary Air Pollutants

Replacing fossil fuel energy sources avoids the release of harmful air pollutants such as nitrogen oxides (NO_x_), sulfur dioxide (SO_2_), and particulate matter (PM2.5). (There are limited studies on reduction in air pollutants from OSW in California, but in the Mid-Atlantic OSW facilities avoid an estimated range of 1.3–6.4 lbs of SO_2_ emissions per MWh and 0.7–1.9 lbs of NO_x_ emissions per MWh [47].) Reductions in air pollutants are site-specific, as they depend on pollution sources in neighboring areas and the extent to which they are curtailed by renewable energy. Relatively little fossil fuel electricity generation exists in California, so the prospects of benefits of this type to the state are relatively small. The Humboldt Bay Power Plants have 10 units with a combined capacity of 304 MW. In Morro Bay, there are no fossil fuel-powered plants in the immediate vicinity. Displacing 5 GW of gas-peaking power plants with 10 GW of OSW capacity would avoid approximately 144 tons of PM2.5, generating USD 19 million in health benefits. (On the Atlantic and Gulf Coast, Shawhan et al. estimate that 32 GW of OSW could prevent approximately 436 premature deaths per year from reduced ground-level PM2.5. When combined with reductions in ground-level ozone pollution, the estimated dollar value of mortality and illness reductions is USD 40/MWh [39].) If California reaches its target of 25 GW by 2045, these benefits increase to USD 48 million. Like the SCC, the value associated with the reduction of ordinary air pollutants will continue to increase.

### 4.3. Job Creation—Annual FTE Jobs

Recent estimates for the co-benefits of job creation and economic impacts from a California OSW industry are in a similar range to those in the original study, with a larger upper bound in all cases. Table 2 presents recent estimates for the economic impacts of OSW projects in California where the sources for each estimate are based on OSW developments of varying capacity. To compare the impacts from each source to the original study, each estimate is derived based on its impact per GW of installed capacity, then scaled to a cumulative OSW capacity of 3 GW for the year 2030 and 10 GW for the year 2040. These estimates are also scaled to a 25 GW capacity for the year 2045, in accordance with California’s AB 525 strategy goals. Results from the original study estimated that a California OSW industry could support between 64,758 and 126,005 job-years between 2020 and 2040 for the construction of 10 GW of OSW capacity. In recent years, estimates of economic impacts for construction exhibit a wide range from 134,697 to 180,267 job-years. Previous estimates for annual job creation from operations and maintenance are 5354 to 6073 for scenarios assuming relatively low levels of support industries operating in California versus high levels, respectively. Recent estimates range from 3833 to 6909 annual jobs.

### 4.4. Economic Growth–GDP Estimates

Results from the original study estimated that a California OSW industry could generate between USD 9.7 billion and USD 18.6 billion in GDP impacts from the construction of 10 GW of OSW capacity by 2040. Recent estimates of GDP impacts from construction are higher, ranging from USD 15.9 billion to USD 21.8 billion. GDP impacts from operations and maintenance in the original study are USD 732 million and USD 838 million for the support industry scenarios, respectively. Recent estimates for GDP impacts from operations and maintenance range from USD 517 million to USD 907 million.

### 4.5. Economic Well-Being—Personal Income

Results from the original study estimated that a California OSW industry could generate between USD 9.3 billion and USD 17.8 billion in direct and indirect personal income from the construction of 10 GW of OSW capacity by 2040. Recent estimates of GDP impacts from construction are slightly higher, ranging from USD 11.3 billion to USD 16.6 billion. Impacts on personal income from operations and maintenance in the original study are USD 689 million and USD 790 million for the lower and higher levels of support industries inputs located in the state, respectively. (The estimates in this section are based on input-output analysis. In this modeling approach, the level of direct and indirect support industries along the supply chain are modeled by adjusting the “regional purchase coefficients” (RPCs), which reflect the percentage of a good or service used in the state that is actually produced in the state (see [10])). Recent estimates for impacts on personal income from operations and maintenance range from USD 313 million to USD 562 million. These gains are lower bounds, as they do not include purchasing power improvements from lower electricity prices associated with OSW. Given the uncertainty about future price differentials, we have chosen not to attempt to measure this consideration.

### 4.6. Equity and Environmental Justice

OSW can advance environmental justice (EJ) by providing clean energy access and affordability, creating high-paying jobs and tax revenues, and reducing pollution in urban areas, which are characterized by a relatively large proportion of underrepresented groups. In general, communities adjacent to fossil fuel power plants can experience adverse health, social, and environmental impacts resulting from everyday operations. Communities surrounding urban ports are particularly vulnerable to high emissions exposures and Superfund sites, while community vulnerability surrounding ports in remote areas is typified by economic concerns, such as long-time unemployment, lower median incomes, and low educational attainment [50]. Displacing gas-fired peaker plants, which are predominantly located in urban areas with environmental justice concerns [51], will create substantial progress in addressing some of these issues. Additionally, OSW can bring economic benefits to lagging areas in California and remote, rural coastal communities. The gains from initial economic investments in both urban and rural settings can be reinvested in community projects and benefits, creating a virtuous cycle for local economies.

Although EJ is discussed as a co-benefit, we are evaluating it in the context of both its positive and negative impacts. The potential for offshore wind projects to advance EJ varies by region and locale, and these come with their own set of context-specific challenges. Furthermore, the benefits of OSW can simultaneously be at odds with other goals within the scope of EJ. Competing goals must be balanced against one another.

Several OSW development areas in California are both within and bordering Native American lands, where Indigenous communities have historically been subjected to environmental injustices. OSW development that does not focus on, or even include tribal sovereignty represents a procedural injustice, threatening to perpetuate the long history of marginalization of these communities (see Section 6.4 for further discussion of tribal EJ issues). Many of the potential negative impacts on Indigenous communities also exist for marginalized communities in urban areas and those surrounding ports where much of the manufacturing, staging, and deployment of OSW will occur.

Disruption of fossil fuel industries by OSW development will harm their host communities economically unless they are replaced by additional employment opportunities. To mitigate these impacts, it is crucial to prioritize an equitable transition by investing in workforce training and education for clean energy jobs, with a focus on the affected communities and workers. In port communities, additional challenges may arise from increased air pollution due to wind-related industrial and trucking/ship activity, along with noise pollution that significantly impacts quality of life.

Moving forward, the EJ co-benefits from OSW will ultimately depend on the extent to which developers and decision-makers engage with vulnerable communities and prioritize positive outcomes for them. The OSW industry cannot undo or avoid root issues that have led to historically vulnerable and underserved communities. However, by acknowledging these problems, this industry can ensure that the benefits produced by OSW activities are strategically invested to create better outcomes. By engaging community representatives throughout the project decision-making process, prioritizing local workforce development, aligning indicators with actionable objectives, and when necessary, blocking harmful project development, the OSW industry can repair broken trust and ensure that the renewable energy transition benefits all communities.

## 5. Key Engineering Challenges

### 5.1. Floating OSW Plant Design

#### 5.1.1. Description

As floating OSW technology evolves and wind turbine capacities exceed 15 MW, the industry faces two primary challenges: (1) developing anchor and cable models tailored to specific seabed conditions, and (2) designing optimal wind farm arrays [52,53]. There are many different anchor technologies, including but not limited to drag embedment anchors, gravity anchors, driven piles, and suction piles. Each type is subject to potential failure mechanisms, so it is critical to design for multiple failure conditions to prevent scouring issues (removal of sediment around tension cables due to wave action) and soil liquefaction issues (shear failure of soil due to liquefaction). The design of mooring lines that connect floating platforms/foundations to the anchors come in many different varieties (e.g., taut or semi-taut, synthetic material, wire rope or steel pipes, and shared between different turbines or attached to just one). Shared mooring lines can reduce installation costs, but have other drawbacks, such as transferring impacts between connected turbine platforms [54].

Further research is needed to test array formations and optimize wind farm array design against different environmental conditions [52]. Optimizing the layout of wind turbines can help mitigate electricity generation losses due to atmospheric wakes, which can reduce power generation up to 38% [55]. (Wakes are regions of reduced wind speed and increased (small-scale) turbulence, which form (regardless of plant design) when the turbine extracts momentum from the wind [56]. Atmospheric conditions have a large impact on how long wakes persist before breaking apart, making them a valuable consideration for the location and layout of wind farms.) Non-uniform blockage effects at the front of wind farms can also impair operating efficiency, particularly as the size of wind farms increases [57]. Cabling for arrays is just as crucial as the design of wind farm arrays. Cabling costs increase with spacing but decrease with larger turbines [58]. Increased distance between turbines minimizes wind speed reductions for downstream turbines, but it also reduces overall efficiency within farms and requires longer subsea cables to connect turbines, thereby increasing production costs. Furthermore, cable burial depth is problematic due to cost considerations; cables can be damaged if they are not sufficiently secured and covered, but greater installation depth is more expensive.

#### 5.1.2. Current Actions

Several solutions are being developed to address these design challenges. Researchers at the National Renewable Energy Laboratory (NREL) are working on a Wind Array Design project to model and design features for all aspects of a wind farm under site-specific conditions [52]. NREL also developed a representative project design envelope (RPDE) containing a comprehensive account of the scale and number of components required for floating OSW farms [59]. Until developers establish detailed project designs, the RDPE will fill a critical gap in the technical specifications and layouts for California’s floating OSW facilities. Meanwhile, the National Offshore Wind R&D Consortium is tracking large grants and efforts dedicated to OSW research in the U.S., identifying approximately 52 projects and 9 specifically dedicated to floating structure engineering (anchor and mooring systems) [53].

Seabed, anchor, and cable models are improving through software platforms that simulate floating turbine performance under various design conditions. Researchers have explored multiple strategies, such as adjusting hub heights, refining turbine arrangements, and creating innovative platform designs. One study identified pentagon-configuration platforms, with turbines positioned at each vertex of the pentagon-shaped foundation, as effective in mitigating wake effects. This theoretical research is promising for optimizing OSW farm designs before substantial investments are committed.

#### 5.1.3. The Future

While significant advances are being made to upscale wind turbines, Musial et al. [53] acknowledge the risks of upscaling turbines too quickly and the need to balance efforts with improving turbine efficiency. To prevent liquefaction and/or scouring, increased R&D on soil types is essential, along with more extensive modeling to determine how specific anchor technologies will perform in various soil conditions. Moreover, developing advanced modeling tools, supplemented by knowledge from other offshore industries, will produce wind farm designs that are better suited to unique environmental conditions and optimized for reliability and efficiency.

### 5.2. Installation

#### 5.2.1. Description

There are four types of floating OSW turbine foundations: spar, tension leg platform, semi-submersible, and barge [60]. In California, the semi-submersible model may be the most economical design for floating OSW farms, but it remains to be seen as newer substructure designs combine positive elements across various foundation types. While there are specific challenges that apply to each foundation design [61], there are a few general challenges, including:

1. Logistics of transporting and erecting turbines: Challenges include extensive drag while towing turbines, distributed buoyancy adding complexity during towing, and downtime losses if maintenance towing to port is delayed [62];
2. Installation of foundations and platforms: This involves tasks such as tensioning anchors, which require large bollards to maintain taut mooring lines [62], and transferring large equipment like generators to turbine structures [60];
3. Working in marine environments: This comes with its own set of obstacles, especially during extreme weather events and wave climates throughout the year, which can delay installation, towing procedures, and maintenance practices [61].

#### 5.2.2. Current Actions

To address the logistics of transporting and erecting turbines, some wind developers are investing in self-propelled modular transporters to assemble turbines without cranes [63]. Various methods are being explored to reduce the challenges of installing floating platforms. One approach involves using floater tensioning devices to cut installation times and costs [60]. Additionally, specialized cranes are in development to ease turbine assembly [63]. Lift vessels with advanced dynamic position systems and features tailored to specific hub heights will enhance safety and efficiency during installation. The OSW industry can mitigate marine environment hazards by drawing on practices from adjacent offshore industries like oil and gas and identifying optimal weather windows for marine operations [64].

#### 5.2.3. The Future

There are potential advantages to floating turbine installation that could lower costs relative to fixed-bottom turbines, such as fewer custom vessels and equipment requirements to handle moorings for floating turbines compared to heavy monopiles or fixed jackets. Improving the installation procedures for floating OSW turbines requires several advancements in mooring and anchoring technologies. Projects like the TAILWIND initiative are developing advanced station-keeping technologies, including dynamic cable systems and innovative mooring designs, essential for maintaining the position of floating turbines in deep waters. Integrating robust simulation and mechanical analysis tools will enhance the reliability and performance of floating systems, ensuring better deployment and operational stability [65].

### 5.3. Operation and Maintenance of Offshore Wind Turbines

#### 5.3.1. Description

Operation and Maintenance (O&M) in OSW farms are critical for ensuring wind turbine efficiency, reliability, and longevity. Robust O&M strategies are required to mitigate risks associated with harsh environmental conditions, such as high winds, saltwater corrosion, and extreme weather [64,66]. The OSW industry faces several challenges in turbine O&M, most notably the high maintenance costs due to the remote and harsh operating environment, the logistical complexity of accessing turbines for repairs, and the need for specialized vessels and equipment [64,66,67]. Environmental factors, such as high winds, waves, and corrosion significantly impact the maintenance of OSW turbines. OSW O&M also demands highly skilled and well-trained personnel to perform maintenance tasks safely and efficiently. Strategies are needed to mitigate potential human errors induced by long shifts, extended periods away from shore, and exposure to extreme weather conditions [68].

#### 5.3.2. Current Actions

The industry has adopted several sophisticated O&M solutions to enhance efficiency and reliability. Key among these is the improvement of crew transfer systems for swift and secure transportation of maintenance staff to and from wind turbines, reducing downtime and expanding the range of weather conditions under which maintenance activities can be conducted. Mother boats provide living quarters for crew members directly at the wind plant, significantly reducing travel times and increasing the window of availability for maintenance tasks. Service Operation Vessels (SOVs) and helicopters for O&M activities are also being explored to improve accessibility and reduce downtime.

Condition-based maintenance (CBM) has emerged as a proactive approach, monitoring the operating state of equipment to determine the optimal timing for repairs, thereby improving wind farm dependability and efficiency [64,66]. Advances in digitalization and remote monitoring technologies have enhanced predictive maintenance capabilities [69]. Furthermore, improved circularity and life-cycle analysis methods, which only target damaged components, can simplify maintenance procedures, reduce new material demand from repairs by 80–90%, and lower carbon emissions from vessel and crane operations by around 80% during repairs. This approach also provides cost savings, limits waste, reduces downtime, and minimizes health and safety risks [70].

#### 5.3.3. The Future

The future of O&M for OSW turbines is poised for increased digitization and automation. Predictive maintenance strategies, advanced analytics, and AI are expected to become more prevalent, predicting failures before they occur to reduce downtime and maintenance costs [45,69]. Integrating digital twins into the O&M process can enable more precise monitoring and maintenance planning [64]. The industry can also benefit from improved forecasting models and tools for better planning and execution of O&M activities, considering the variability of marine conditions [71].

Over the next few years, the OSW industry will likely focus on developing more durable turbine components and innovative designs that require less maintenance. O&M practices will also place a greater emphasis on minimizing environmental impacts on marine ecosystems. As autonomous drones and underwater vehicles become available for inspection and maintenance tasks, human intervention could be reduced, improving overall safety. Advances in materials science may lead to more corrosion-resistant components, further reducing maintenance requirements [64].

### 5.4. Decommissioning

#### 5.4.1. Description

Significant advances have recently been made in both research and practice addressing the decommissioning and disposal of OSW components. Much of this research focuses on circular economy initiatives aimed at reducing material waste and minimizing the impacts and costs associated with the demand for raw materials and their disposal. These initiatives could reduce carbon emissions from the offshore wind sector anywhere from 6 to 34% through improved material efficiency and waste reduction [72]. Moreover, reducing new raw materials could avoid up to 2.6–3.6 GT CO_2_e emissions from OSW deployment between 2020 and 2040 [73].

The primary strategies for increasing circularity in the OSW industry involve End-of-Life (EoL) management approaches that maximize the use of functioning assets while delaying full decommissioning for OSW units nearing the end of their design life (25–30 years). These approaches include full repowering, partial repowering, and life extension, and their selection is determined by a Lifetime Technical Evaluation that assesses the integrity of each asset [70]. Partial repowering exhibits the most potential, producing significant positive cash flows for up to 15 additional years while minimizing reinstallation costs and material waste, and maximizing the lifetime use of foundations, generation, and transmission assets. Life extension is also attractive, with minimal reinstallation costs and increased positive cash flow for up to 10–15 years [74].

Eventually, asset materials will need to be decommissioned, with incineration or landfilling as the worst-case scenario. At this stage, technologies and methods for recycling turbine components into useful secondary products are crucial. (Different component groups/material types include steel (contributing to 70–90% of turbine mass), mechanical components (consisting of alloys made of several different types of critical metals and used in gearboxes, bearings, etc.), electrical components (including permanent magnets which rely on Rare Earth Materials with high magnetic energy density, and which are acutely environmentally harmful to produce), and composite materials used in turbine blades (consisting of difficult-to-separate polymer resin matrices with reinforcing glass/carbon fibers, high-density foam or balsa wood cores, and metal-based coatings) [70].) Bennet and Spyroudi [70] conclude that 85–90% of a turbine is technically recyclable. Each major component group and material faces unique challenges for achieving zero waste, but there is potential for substantial environmental and economic benefits by reducing raw material demand and creating high-skilled jobs in refurbishment, remanufacturing, and recycling.

#### 5.4.2. Current Actions

Companies worldwide are developing circularity solutions to address technical and logistical decommissioning challenges. The major challenges in recycling materials include separation, recovery, and reprocessing. One approach to simplify these processes is to implement design changes that make materials inherently more separable or recyclable. For example, some original equipment manufacturers (OEMs) are exploring alternative bio-based resins made from natural polymers, which melt at lower temperatures, allowing component materials and fibers in turbine blades to be more easily separated. NREL researchers are also developing wind turbine blades out of plant-based materials that can be recycled [75]. Most wind turbine blades are not recycled, and by 2050 estimates suggest more than 43 million tons of landfill waste could be generated globally from turbine blades [75]. Moreover, these bio-based materials address more fundamental microplastic and pollution issues that arise from recycling synthetic, petroleum-based materials [76]. Through these efforts, two major lessons have emerged. First is the advantage of developing domestic recycling solutions to prioritize local job growth and save time and costs, and to reduce emissions associated with exporting scrap and re-importing remanufactured materials. Second, it is generally easier to deal with pollution issues by addressing them from the start with sustainable material selection rather than making costly pollution containment measures later in the product lifecycle [74].

#### 5.4.3. The Future

EoL strategy for OSW farms is determined by factors such as fatigue loads, seabed conditions, and operational asset integrity, all influenced by decisions in the early stages of design and development. With proper planning and implementation, wind farm lifetimes can be extended by up to 50%. Therefore, incorporating EoL strategy planning early in OSW projects is crucial to optimize performance, maximize asset use, and minimize waste. For fully decommissioned turbines, operational sub-components should be separated from metal waste, refurbished, and sent for reuse as replacement parts in other wind farms, or repurposed to create new products in other markets [77].

To build industry confidence in refurbished materials, several companies are advocating for government-led regulations and standardized certifications, such as an ‘As New Warranty’. These measures could strengthen trust and partnerships between remanufacturers and their customers, enhancing transparency and communication regarding the quality of recycled materials. Establishing widely accepted industry standards would also enable the scaling up of recycling infrastructure to meet the growing market demand. In alignment with this demand, OEMs should reserve virgin materials for high-tensile applications while utilizing recycled materials for less demanding uses, as recycled materials are typically of lower quality than their virgin counterparts [78]. Finally, many companies struggle with market penetration due to a lack of industry knowledge and connections with key operators and decision-makers in the OSW sector, despite possessing the necessary technical skills and technologies to implement these solutions. Bridging this gap should be a priority for the government and OSW industry to pave the way for a more self-sustaining circular economy [70].

## 6. Key Broader Challenges

### 6.1. Transmission (Sea and Land)

#### 6.1.1. Description

The large buildout of transmission needed to connect California’s proposed OSW farms to the electricity grid will require expeditious planning, approval, siting, permitting, and construction of multiple long-distance high-voltage transmission lines. This is particularly challenging on the North Coast, where the current infrastructure is substantially undersized to meet large-scale OSW loads. To meet increased load demands, California OSW will require substantial upgrades to the land-based transmission network as well as offshore transmission solutions, which are constrained by backlogged engineering studies and permitting delays.

For offshore cabling, this problem is exacerbated by usage conflicts between the U.S. Department of Defense operational areas, vessel traffic, fairway designations, cable landing locations, existing submarine cable locations, fishing grounds, and marine protected areas. Transmission buildout must overcome complex geophysical characteristics—submarine canyon crossing issues for laying cables, turbidity flows, fault lines/seismic displacements, steep slopes, and substrate conditions. Furthermore, there are problems associated with cable landfall challenges, such as road access, deepwater ship access, and potential intrusion into protected marine habitats [11].

#### 6.1.2. Current Actions

A Memorandum of Understanding (MOU) signed by the CEC, CPUC, and California Independent System Operator (ISO) in 2022 will enhance coordination for resource and transmission planning, interconnection processes, and resource procurement [2]. This will ensure future transmission needs are met through California ISO’s annual Transmission Planning Process and their 20-Year Transmission Outlook. In its latest 2023–2024 TPP, the California ISO has approved more than USD 4.5 billion for transmission to connect OSW farms from the North Coast to major hubs of demand in other parts of the state [79]. Private companies are also coordinating their material and planning needs further in advance to overcome the growing demand for procurement and installation of transmission networks while creating opportunities for shared transmission systems, eliminating the need for new cables and transmission infrastructure entirely [80]. As developers connect to and construct these networks, they are increasingly relying on more advanced geophysical surveys, monitoring systems, remote-operated vehicle (ROV) inspections, and horizontal directional drilling methods to overcome many of the technical barriers and environmental concerns.

#### 6.1.3. The Future

Coordinated transmission planning and clustered development of offshore transmission networks can further mitigate the current inefficiencies and delays of the project-by-project approach to transmission buildout [81]. Clustering increases the economic feasibility of OSW by enabling transmission infrastructure sharing, providing higher capacity with fewer total cables, greater access to remote offshore locations, and reduced environmental impact [82,83]. A planned power corridor approach with shared cable routes and landing points can streamline permitting and engagement compared to multiple individual radial connections [81]. The National Interest Electric Transmission Corridor (NIETC) Designation has also identified major transmission corridor opportunities that can unlock federal financing tools and expedite permitting and siting of transmission lines [84]. Reconductoring existing transmission lines with advanced conductors can double transmission capacity along existing corridors, bypassing some of the constraints of building new lines [85]. Lastly, preliminary studies indicate the potential for optimized ocean grid infrastructure or offshore wind transmission networks to improve transmission investments. Such technology could streamline interregional planning across Washington, Oregon, and California [86].

### 6.2. Seaport Capacity

#### 6.2.1. Description

California can only realize its OSW potential with adequate seaport infrastructure, but most of the state’s existing ports are inadequate for commercial-scale floating OSW buildout. Seaport capacity is primarily limited by port depth, which will require costly dredging expenditures to meet the requirements for staging and transporting turbines. OSW seaports require three primary types of port sites—staging and integration (S&I), operation and maintenance (O&M), and manufacturing and fabrication (M&F)—which altogether require upwards of 35–200 acres and minimum dredging depths of nearly 40 feet [87,88]. The funding and time required to establish this are substantial. Shields et al. [50] note that an individual S&I port site costs an estimated USD 1 billion, with a construction timeline of around 10 years. Moreover, achieving California’s 25 GW OSW target by 2045 will require four staging and integration sites and at least eight operation and maintenance sites, with an estimated USD 11–12 billion in port upgrades [50].

A dedicated fleet of vessels will also need to be developed in parallel. However, supplies are limited, and the use of foreign ships is barred by the Jones Act, which restricts the transportation of goods by water in the US to US-built and US-registered vessels. In addition to a shortage of shipyard availability for constructing Jones Act-compliant vessels, vessel production is further constrained by a lack of long-term contracts, high build costs, and stringent regulations by the California Air Resources Board [89]. An insufficient supply of new vessels could create a tremendous pinch point in deployment, thereby stalling California’s OSW planning goals.

#### 6.2.2. Current Actions

The California State Lands Commission has identified and assessed a short list of existing and greenfield port sites that could meet the requirements for OSW S&I, O&M, and M&F [89]. Among the sites identified, investments are already moving ahead in the Port of Humboldt Bay and the Port of Long Beach. In 2022, the CEC awarded a USD 10.5 million grant to the Port of Humboldt for infrastructure supporting OSW projects [90]. This was followed by a USD 8.6 million grant in 2023 from the U.S. Department of Transportation (DOT) for a marine terminal and bay-wide master plan project. Most recently, Humboldt has received USD 426.7 million from the US DOT through its Nationally Significant Multimodal Freight & Highway Project (INFRA) grant program, which will fund the infrastructure to support OSW farms [91]. In Long Beach, the Port is moving ahead on a proposed 400-acre terminal for OSW turbine assembly, although it is still raising funds for the USD 4.7 billion project [92]. Additional funding for Long Beach and Humboldt is likely to come from the state’s recently approved SB 867/Proposition 4, a USD 10 billion bond act containing USD 475 million to help pay for the expansion of OSW ports [93].

#### 6.2.3. The Future

Developing an efficient West Coast port network will require effective communication and collaboration, increased funding and incentives, and increased workforce capacity. New training programs will be needed to attract and develop the workforce for M&F, S&I, and O&M needs. An intergovernmental steering committee could develop and maintain communication channels, encourage engagement with local and tribal communities, and facilitate the distribution of benefits to communities hosting OSW development. Regional port collaboration could also optimize efficiencies in the broader region and ensure ports effectively support next-generation wind turbine systems [82]. Early engagement with project developers and relevant groups will be critical to efficiently review permits for port development and minimize port congestion as projects move forward. California has already initiated an in-state port planning process under AB 525 to facilitate this kind of collaboration. This process could be improved by including decision-makers from Oregon and Washington.

The OSW industry should take a more proactive approach to supplying Jones Act-compliant vessels that meet CARB’s environmental regulations [89]. Increasing coordination will be essential to leverage expertise and capacity, and generate alternative funding schemes to de-risk investments in vessels and extend tax credits to support their long construction timeline. Backstop revenue guarantees could also ensure private investment while covering the risk that new ships and new port terminals may not be fully utilized. Finally, investments in West Coast ports should take every opportunity to grow the green economy, stabilize affordable housing, and improve air quality through the decarbonization of port activity [82].

### 6.3. Environmental and Wildlife Concerns

#### 6.3.1. Description

There are six potential adverse effects on the environment and wildlife due to OSW farms, including alterations to the ocean and the surrounding atmosphere, electromagnetic field (EMF) radiation, habitat alteration, underwater acoustic disruptions, structural wildlife impacts, and water quality changes [94]. Oceanic changes occur because of wind wakes formed by moving turbine blades [95], which may reduce upwelled nutrient-rich waters transported to coastal zones and create uncertain impacts on coastal ecosystems reliant on those nutrients [94,96,97]. Atmospheric changes result from reductions in regional wind speeds, which are estimated at 5% [98,99]. EMF radiation can potentially disrupt the natural EMFs that some marine species utilize for navigation, communication, and mating purposes. In some cases, these magnetic fields can affect the growth of an animal, while other animals benefit by using EMFs as environmental cues [100].

Introducing new OSW structures into marine environments could facilitate colonization by invasive species that alter existing habitats and ecosystems [101]. All aspects of developing an OSW farm generate underwater noise impacting marine life, from developing an initial topographic survey of the area, to installation processes for offshore floating foundations and anchors, to operation and maintenance activities that use large vessels, to decommissioning processes [102]. Vibrations can also induce sediment shifts that potentially harm marine life [103]. Leaks or spills of oils, chemicals, paints, etc. used in turbine construction and maintenance can contaminate and degrade water quality in the surrounding marine environment [94]. Finally, turbine blades remain a potential threat to both birds and bats [104].

#### 6.3.2. Current Actions

BOEM has declared a notice of intent (NOI) to prepare a programmatic environmental impact statement (PEIS) for the five leased areas off of Humboldt and Morro Bay [88]. BOEM is collaborating with the National Oceanic and Atmospheric Administration (NOAA) to protect aquatic animals from OSW farms, creating strategies to mitigate potential effects on endangered species of whales [105]. Wind developers are also coming up with strategies to mitigate potential environmental effects during all stages of the life cycle. Rockwood et al. [106] identify areas for optimal OSW siting while preserving existing ocean uses and protecting the marine and coastal environments. Weather radar is being used to identify the migratory patterns of birds and better determine sites for OSW farms [107]. Dominion Energy, the utility behind Coastal Virginia Offshore Wind, is actively monitoring wildlife activity during the foundation installation process and pausing activity when animals are detected in the area [108].

#### 6.3.3. The Future

Although many of the environmental impacts of OSW are yet to be observed, most, if not all, of these challenges can be actively addressed. Comprehensive R&D is needed to assess the potential impacts on fisheries, coastal processes, and other sectors from changes in upwelling, nutrient transport, and wind speeds caused by large OSW deployments [109]. Mitigating EMF impacts requires careful design of subsea cable routes, burial depths, shielding, and cable types to minimize exposure to sensitive marine ecosystems [110]. Ecological modeling is necessary to characterize biodiversity and species relationships with OSW infrastructure to mitigate habitat impacts [95].

While current data do not indicate that underwater noise significantly harms aquatic life, additional monitoring is essential, especially for behavioral reactions to underwater noise [109]. Wind developers are actively studying underwater noise mitigation during the installation phases, which are typically the most disruptive [111]. Implementing deterrent methods and collision risk modeling in turbine design can help minimize impacts on animal populations. For example, reducing turbine rotational speeds can decrease bat and bird collision rates [112]. Additionally, using non-toxic, marine-safe materials, already proven in other offshore industries, can mitigate water quality risks.

### 6.4. Indigenous and Fishing Industry Concerns

#### 6.4.1. Description

OSW development in California has raised concerns regarding the involvement of Indigenous tribes and local community organizations, as well as the potential impacts on local fishermen. Many Indigenous communities rely on OSW areas for economic, cultural, spiritual, and food-related security, so it is critical to address these conflicts and find ways to minimize the impacts without stifling the industry’s growth. Some tribes also have historical connections and treaty rights to natural and cultural resources, access to which may be threatened or obstructed by the proposed OSW areas. When tribes were removed from their ancestral lands in the 18th and 19th centuries, treaties were established to preserve their rights to access and utilize fishing grounds, many of which include offshore areas [113]. Meanwhile, OSW sites in commercial fishing operation areas create potential restrictions for fishing access to leased waters, adversely affecting fishermen, increasing time and cost, and disrupting fishing port infrastructure [114,115]. Evidently, the impacts of OSW do not represent a just transition by default.

#### 6.4.2. Current Actions

The National Congress of American Indians and several California tribes (the Chumash, Yurok, and Tolowa Dee-ni’ tribes have all released statements in various outlets to address the conflicts between OSW projects and indigenous interests) have called for a halt on OSW development until a transparent process is established that respects tribal environmental and sovereign interests [113,116–118]. Despite ongoing concerns, there are several positive signs from recent research efforts, local collaboration and initiatives, as well as state and federal action. A joint position by the Northern Chumash Tribal Council and Morro Bay Offshore Wind Leaseholders will enable the co-existence of a national marine sanctuary in Morro Bay alongside OSW development areas [119]. Tribes in California and across the country are beginning to express interest in collaborative investment OSW partnerships in an effort to establish mutually beneficial opportunities for OSW development [22]. The Humboldt Area Foundation is also implementing the Redwood Region Climate & Community Resilience Hub (CORE Hub) to promote an “equitable buildout” of OSW energy [120].

At the state level, California’s Offshore Wind Expediting Act (SB 286) will create a new fisheries working group, designed to develop best practices for communication, socio-economic impact analysis, data collection, adaptive management, and compensatory mitigation for unavoidable tribal and fishing impacts [121]. These strategies will be binding for any applicants seeking permits for OSW projects moving forward. The Biden Administration has also released a U.S. Ocean Justice Strategy that will embed ocean justice in federal activities, develop a diverse, equitable, inclusive, and accessible federal ocean workforce, and enhance ocean justice through education, data, and knowledge [122].

#### 6.4.3. The Future

As more OSW projects are developed or proposed on or near sensitive tribal lands or fishing communities, it is imperative to involve the perspectives of those with direct and intimate knowledge of the local context. Respecting California’s recognition of tribal sovereignty and leveraging Indigenous knowledge is critical for the success of OSW deployment [22,123]. Meaningful involvement of Indigenous communities, considering multi-faceted aspects of justice, can help reconcile abusive histories of fossil fuel development while aligning future energy production methods with Indigenous values of land and resource use [124]. An inclusive process that benefits local communities is also necessary to maintain the ethical integrity of projects, generate community support, build resilience, and protect local ecosystems and natural resources [125].

Community Benefit Agreements (CBAs) will be an essential tool for providing economic empowerment mechanisms tailored to local communities [126]. As a multi-year endeavor, CBAs must be initiated early in the development process to align with OSW project timelines and achieve mutually beneficial outcomes for communities and developers alike. Project Labor Agreements (PLAs) can also create additional provisions for targeted local hiring that support workforce development and retain economic benefits within local regions [120]. Both CBAs and PLAs have been included in BOEM’s leasing process. Prioritizing these measures early and diligently in the OSW development process will help address Indigenous and fishing industry concerns while ensuring equitable outcomes that advance environmental justice.

### 6.5. Supply Chain

#### 6.5.1. Description

California lacks the supply chain, manufacturing capacity, and qualified workforce to meet its OSW targets, adding cost and risk pressures to planned and ongoing projects. Many domestic suppliers are holding out for a more stable and clear growth outlook before making commitments to manufacturing investments, necessitating imports from European and Asian suppliers for the majority of OSW projects. The instability of recent offtake contracts on the East Coast has kept manufacturing supply chain capacity in a further state of limbo. Globally, the widespread expansion of OSW is straining supply chains, adding to inflation-ridden project costs.

A West Coast supply chain could reduce travel times, lower transportation emissions, and may decrease project costs. It could also provide employment benefits, estimated at over 30,000 full-time positions by 2035 [127]. While the domestic workforce required to construct, operate, maintain, and, more broadly, reach OSW development targets is inadequate, many communities would benefit immensely from the economic opportunities OSW could provide. Community vulnerabilities such as linguistic isolation, low educational attainment, and long-time unemployment create workforce accessibility challenges that need to be overcome to provide better access to these opportunities and ensure the economic benefits from OSW reach the communities that need them most [50]. This is particularly important for port communities, which will bear the burdens associated with the OSW development due to their proximity to port activities.

#### 6.5.2. Current Actions

While the U.S. remains reliant on imported wind turbine components, investments in domestic OSW factories are rising as East Coast projects progress and in anticipation of lower interest rates on the horizon [128]. The Inflation Reduction Act has also renewed confidence in supply chain expansion [129]. From 2021 to 2023, the Oceantic Network [25] identified a total of USD 7.7 billion in declared supply chain investments. More funding will be needed, however. Shields et al. [127] have created a detailed supply chain roadmap, estimating that building a robust domestic supply chain by 2030 will require USD 22.4 billion in supply chain investments and at least 34 new manufacturing facilities. (This does not include investments for support vessels, workforce training, or expansions of existing businesses. Total investments across the industry are likely much higher.) To continue expanding this supply chain nationwide, O’Boyle et al. [130] estimate that cumulative investment will need to surpass USD 260 billion through 2045. To address gaps in California’s OSW workforce, Fox and Lehmann [131] have conducted a comprehensive assessment of workforce requirements, with recommendations to develop training programs, align investments with regional port strategies, and collaborate with unions, trade organizations, and educational institutions. Together, these efforts can support local communities and supply the necessary components that will make this industry possible.

#### 6.5.3. The Future

To meet the growing pipeline of OSW demand, future investments should target key enabling infrastructure, prioritizing supply chain security, project efficiency, and local economic development [22]. West Coast states should continue evaluating how regional supply chain collaboration and local industry clusters can mitigate global supply chain risks while creating broad-based economic and employment benefits, particularly for historically and systemically underserved communities [50]. Initiatives like the Federal-State Offshore Wind Implementation Partnership and the Pacific Offshore Wind Consortium can address persistent supply chain gaps through their support for research and innovation, workforce education, professional development, and coordinated knowledge exchange [132,133]. States should also consider flexible requirements for local content sourcing to ease constraints on short-term OSW development until a sufficient domestic supply chain develops [134].

### 6.6. Market Challenges

#### 6.6.1. Description

Technical, microeconomic, and macroeconomic challenges have created uncertainty, rising costs, and funding gaps, resulting in project delays and cancellations for several OSW projects. The risks and vulnerabilities associated with these early commercial deployments will continue being pushed to future projects until sufficient demand certainty and reliable project delivery are achieved. As an industry in its early stages, OSW is already vulnerable to exogenous risks and depends on effective risk mitigation to overcome first-mover challenges and existing market turbulence. Furthermore, inconsistency and lack of transparency in procurement schedules and requirements constrain the capacity of developers for long-term planning and investing [22].

#### 6.6.2. Current Actions

The delays and cancellation of projects on the East Coast are being addressed in part through new procurements (“re-bids”) and revised projects that are amenable to the current market conditions, with additional commitments to ensure the necessary infrastructure and supply chain mechanisms are in place to facilitate OSW deployment [22]. Not only are states soliciting new offtake contracts, in aggregate they are increasing overall OSW demand while providing improved contract terms [25].

The federal government has also made headway in creating a dependable pipeline of projects through permitting and approval of OSW projects. In addition to new permits, concerns over short-term and unpredictable tax credits are being addressed through the Inflation Reduction Act, which decreases restrictions on tax credit transferability, thereby increasing investors in OSW projects and simplifying transactions [135]. Furthermore, the passage of AB 1373 in California will provide the necessary coordination and regulatory authority for establishing large, long-term electricity contracts that are critical for OSW projects [136]. In August 2024, the CPUC approved a decision to authorize central procurement of up to 7.6 GW of offshore wind, with solicitations beginning ~2027, for projects coming online in 2035–2037. This central procurement process, as well as how the CPUC values the benefits of offshore wind in judging the cost-effectiveness of contracts, will be essential to the success of this industry.

#### 6.6.3. The Future

Moving forward, the emerging OSW industry will depend on continued support and collaboration with government institutions to de-risk projects and address funding gaps across the industry. Stronger demand signals are needed. Accordingly, Ury et al. [22] note that states should help clarify expectations, de-risk project-scale financing, ensure future deployment is better informed, accelerate re-bid solicitations, and set clear procurement schedules.

### 6.7. Other Concerns

Conflicts between OSW, military, and aviation operations have been an ongoing concern for the Department of Defense and the Federal Aviation Administration given that wind farms can interfere with radar systems and physically obstruct areas needed for training, testing, and general national security measures [137–139]. Ongoing discussions have established a mutual understanding of the need for balancing sustainable energy solutions with military operating areas in California [140]. In August 2023, the U.S. Department of Defense reached a mitigation agreement with CADEMO for floating OSW projects near the Vandenberg Space Force Base [141]. This agreement includes a series of de-confliction protocols for wind turbines operating amidst Vandenberg’s space launches and represents the first agreement between OSW development and the U.S. military on the West Coast. While there are ongoing technical efforts to mitigate interference concerns [142,143], many of the solutions will require legislative changes to empower state and federal bodies to coordinate across military operations and flight paths [144].

Opposition has emerged across the U.S. in response to all kinds of renewable energy developments, including OSW. The sentiments and actions are extensive, taking shape in local restrictions, state-level restrictions, and contested projects [145]. In several high-profile cases in the northeast, efforts to block OSW projects are backed by fossil fuel interests, climate denial think tanks, and community groups [146]. Misinformation often emerges as a result, such as the rumors that OSW turbines kill whales [147], skewing public perceptions and catalyzing opposition against current or future developments. As much as the deployment of OSW is a technical challenge, it is also a challenge of public relations. Advocates, policymakers, and developers will be forced to contend with the many forms of renewable energy opposition and NIMBY-ism that can threaten the success of California OSW. Furthermore, Changes to the national political climate from the 2024 election could drastically impact current investments in OSW and other renewable energy [148]. The election results may prevent new entries into the California OSW market [25]. (It should also be noted that all fuels and energy technologies, both non-renewable and renewable, receive government subsidies [149]).

## 7. Discussion

### 7.1. Current Status and Future Imperatives

Since the original study by Rose et al. [10], which was based on data for 2021 and prior years, OSW has faced some economic headwinds that temporarily constrained its value proposition for California’s electric grid. However, OSW still exhibits several advantageous system, economic, and environmental attributes that are expected to improve as the industry matures. Recent increases in costs are likely to be offset by OSW deployment experience, supply chain and infrastructure improvements, and government policy support. In terms of co-benefits, the reliability of OSW is improving with advances in wind turbine technology and battery storage. In terms of environmental co-benefits, OSW can play an instrumental role in replacing natural gas plants, preventing land intrusion, and advancing environmental justice. Lastly, OSW continues to provide substantial job creation co-benefits, estimates of which have increased in recent studies.

Although there are many differences between the OSW and offshore oil and gas industries, they share similar human, organizational, and technological (HOT) subsystems that provide valuable insights into the safety and risk management for future deployment of any offshore technology, including OSW [150,151]. Poor safety culture and safety management systems (SMS) are typically the underlying cause of major offshore oil and gas accidents, manifested “through failures in the design phase, failure to identify hazards, [and] unsafe operations or lack of adequate response procedures” [152]. These findings are corroborated by recent studies of systemic risk in the offshore oil and gas industry, which found that people (i.e., human behavior side of offshore activities), human-systems integration (HSI, i.e., how the people at all levels are organized, motivated, trained, and managed to carry out their assigned work effectively), and systems (i.e., design and hardware of physical systems, and regulatory environment) are the three primary domains of risk control [153]. OSW developers should consider incorporating these lessons in their own operations to ensure that human factors help, rather than hinder, the industry’s progress.

Successfully developing OSW in California and capturing its myriad benefits will ultimately depend on how broader challenges and pitfalls are addressed. Tables 3 and 4 present a summary of these challenges and their potential solutions. Effective transmission buildout will require more advanced interregional transmission planning, streamlined coordination, and improved permitting frameworks. Overcoming inadequacies in the supply chain depends on significant port infrastructure upgrades, vessel construction and repurposing, expansion of domestic component manufacturing, and consistent government support. Government support and industry collaboration are also needed to address community vulnerabilities and cultivate a robust workforce to support a California OSW industry. Increased stakeholder partnerships are required both to garner support for OSW projects and to ensure their associated benefits are distributed equitably within and across local communities. Lastly, identifying mutually beneficial project agreements is critical to achieving a sustainable clean energy industry.

While it is imperative for the success of the OSW industry to rigorously evaluate, consider, and address all of the challenges and pitfalls characteristic of this technology, it is also important to acknowledge that no solution is perfect. Such challenges should not discourage investments in the energy transition and efforts to rapidly test and deploy renewable energy technologies. It is also important to consider that other energy sources, especially the fossil fuel sources that OSW would aim to displace, come with their own set of significant drawbacks. It is essentially a question of which alternative strikes a better balance. In light of the current evidence to support OSW, and given the urgent need to rapidly decarbonize the electric grid, it is clear that OSW has the potential to offer far more benefits than drawbacks if it is implemented and planned correctly and holistically.

### 7.2. Limitations of the Analysis

One of the major limitations of any analysis of floating OSW is the dearth of construction and operating experience. We have endeavored to overcome this limitation by identifying consensus assessments from a variety of experts (as in the case of LCOE estimates in Figure 1) and referencing experience with technologies that have similar characteristics (as in the discussion of human factors for offshore oil and gas rigs). Several of the basic conditions of OSW operation are more reliable than others, such as the fact that the operation of the technology does not generate GHGs (except for its construction and upstream supply chain fabrication of system components), or the manpower estimates for construction and operation. We have not measured these, but it should be noted that all technologies generate GHGs, as well as other pollutants, along their supply chains. Still, important estimates for which the aforementioned serve as the basis are subject to much greater uncertainty, such as the extent of displacement of fossil-fuel electricity generation and the social cost of carbon, or the multiplier effects of direct employment in relation to the extent of location of support industries within California.

The sources cited on the economic impacts of OSW are compared to the previous study by extrapolating their impacts on a scaled per GW capacity. As such, these are simply proportional estimates that do not consider how impacts change based on project scale; however, our linear extrapolation errs on the conservative side, as economies of scale are very likely to ensue. There are also limitations to the accuracy of comparisons and potential inconsistencies in methodology or assumptions across the studies that were analyzed. Furthermore, rapidly changing political, technological, and market conditions in the OSW industry mean that, while these snapshots of current conditions can be considered with some confidence, future projections are still uncertain.

The dynamic nature of the OSW industry also influenced the scope and extent of available literature analyzed. For relevant issues that lack peer-reviewed research, this paper relied on a variety of grey literature sources that have standard limitations, but we were careful to evaluate those from more reliable sources. Still, many of these analyses are informed primarily by academic research and have limited input from key stakeholders in the OSW industry, including developers, operators, manufacturers, policy makers, and host communities.

## 8. Conclusions

This paper provides an updated assessment of the potential benefits and challenges of OSW electricity in California. Through an extensive synthesis of the literature, expert interviews, and additional calculations by the authors, we conclude that OSW has a bright future. It adds to the mix of technologies that can reduce GHGs and other forms of pollution, promoting the overall sustainability of electricity service provision. Expert consensus indicates that OSW is on the verge of being cost-competitive with most other renewable energy technologies and can improve electricity system reliability, especially in combination with solar energy. Attaining larger scale of development will be crucial to achieving this competitive threshold. Furthermore, there is strong reason to believe that costs will decline through production, economies of scale, and deployment, as witnessed in other clean energy technologies.

The economic benefits of OSW include substantial contributions to equity by offering employment opportunities in lagging rural regions. Environmental justice benefits are derived from ordinary air pollutant reductions in urban areas, where most remaining fossil fuel electricity generating installations are located. Several other relative benefits are enumerated in the paper as well.

OSW is not, however, without its engineering and broader challenges. We have outlined many of these, providing background, current status, recent changes, and future outlooks for each. The bottom line is that design improvements, best practice operating experiences (including lessons from operating experiences of similar technologies), and stakeholder involvement in the siting, construction, and operation of OSW facilities can overcome most of the obstacles and shortcomings of this technology.

Public and private sector decision-makers in California have clearly established an ambitious statewide development target of 25 GW of OSW capacity by 2045. A host of uncertainties face the industry, including political considerations, which we have not speculated on, in favor of sticking to facts and expert opinions on the direct and broader ramifications of OSW construction and operation.

While planning and operating experience will provide some of the greatest insights to advance OSW in California, the industry can also benefit from future research in several key areas. First, the extent of additional benefits from meeting the surging demand for electricity driven by data centers, artificial intelligence, electric vehicles, and electrification of the built environment. Second, research on strategies and complementary approaches to reduce OSW operating disruptions and enhance overall system reliability. Lastly, as climate impacts become more severe, energy infrastructure in California and across the globe will experience heightened risks. More research into the individual and system level resilience aspects of OSW will therefore be valuable to enhancing the industry’s future viability.

Overall, we conclude that OSW is an increasingly attractive electricity generating technology, and the conditions in California are well suited to its development. While there are headwinds to bringing this technology to scale, the potential benefits are worthy of pursuit. If climate policy continues to gain momentum in the state and across the U.S., OSW should be considered an integral part of the solution.

## Figures and Tables

**Table 1.** GHG Reduction Benefits (million 2023 USD).

	10 GW OSW (2040)			25 GW OSW (2040)		
	
	Lower Estimate	Middle Range	Upper Estimate	Lower Estimate	Middle Range	Upper Estimate

**Original Study (Annual)**	USD 12	USD 55	USD 97	-	-	-
**Updated Benefits (Annual)**	USD 240	USD 381	USD 1169	USD 600	USD 953	USD 2923
**Updated Benefits (Lifetime)**	USD 6846	USD 10,658	USD 32,700	USD 17,116	USD 26,645	USD 81,750

**Table 2.** Economic Impacts of OSW Projects in California.

Phase	Impact Indicator	3 GW OSW (2030)		10 GW OSW (2040)		25 GW OSW (2045)
	
		Original Study	Update	Original Study	Update	Update

**Construction**	**Employment (job-years)**	22,049–42,923	40,409 ^a^–54,080 ^b^	64,758–126,005	134,697 ^a^–180,267 ^b^	336,742 ^a^–450,668 ^b^
	**GDP (million 2023 USD)**	3313–6338	4758 ^a^–6538 ^b^	9739–18,622	15,859 ^a^–21,793 ^b^	39,646 ^a^–54,482 ^b^
	**Personal Income (million 2023 USD)**	3154–6043	3396 ^a^–4980 ^b^	9271–17,754	11,320 ^a^–16,599 ^b^	28,301 ^a^–41,497 ^b^

**Operation and Maintenance**	**Employment (annual jobs)**	1375–1560	1150 ^a^–2073 ^a^	5354–6073	3833 ^a^–6909 ^a^	9583 ^a^–17,273 ^a^
	**GDP (million 2023 USD)**	188–215	155 ^a^–272 ^a^	732–838	517 ^a^–907 ^a^	1292 ^a^–2268 ^a^
	**Personal Income (million 2023 USD)**	177–203	94 ^b^–169 ^a^	689–790	313 ^b^–562 ^a^	782 ^b^–1405 ^a^

a [48]

b [49].

**Table 3.** Key Engineering Challenges.

Aspect	Challenge	Example Solution
Design	Anchor and cable model connections Optimizing wind farm arrays	Turbine performance simulation Enhanced platform configuration
Installation	Turbine transportation and erection Harsh marine environments	Specialized lift vessels and cranes Weather monitoring and forecasting
Operations & Maintenance	Logistical complexity accessing turbines High maintenance costs	Enhanced crew transfer systems Condition-based maintenance and life-cycle analysis
Decommissioning	Cost effective recycling Repowering Life extension	New material applications and innovations Lifetime technical evaluations and extensions ‘As New Warranty’ product regulations

**Table 4.** Key Broader Challenges.

Aspect	Challenge	Example Solution
Transmission	Planning, permitting, and development delays Usage conflicts Complex geophysical conditions	Long-range planning, clustering, and reconductoring Ocean grid networks Advanced surveying, horizontal directional drilling
Seaport Capacity	Inadequate port infrastructure Insufficient supply of seaport vessels	Intergovernmental port investments and guidance Vessel investment de-risking
Environment & Wildlife	Ocean and atmosphere changes Electromagnetic field radiation Habitat alteration Acoustic disruption Structural wildlife impacts Water quality changes	Comprehensive R&D assessments Subsea cable design improvements Enhanced ecological modeling Acoustic monitoring and mitigation strategies Deterrent methods and collision risk modeling Non-toxic marine-safe materials
Indigenous and Fishing Industry	Tribal engagement and conflicts with cultural sites Adverse effects on local fishermen	Early, frequent, transparent stakeholder engagement Community benefit and project labor agreements
Supply Chain	Lack of in-state manufacturing Workforce gaps and accessibility issues Strained global supply chain	Regional supply chain collaboration and investments Targeted training programs and incentives Flexible sourcing requirements
Market Challenges	Offtake contract cancellation Procurement schedule consistency and transparency Centralize procurement needs	Re-bid solicitations, de-risk project-scale financing Expand procurement schedules and transparency AB 1373 central procurement process
Other Concerns	Conflict with military training sites and operations Misinformation and local opposition	Coordination and de-confliction protocols Outreach and education campaigns
